# Concurrent validity between self-reported International Physical Activity Questionnaire Short Form and Fibion accelerometer data among young adults in the UAE

**DOI:** 10.1186/s40001-024-01975-5

**Published:** 2024-08-19

**Authors:** Ashokan Arumugam, Nour Alsaafin, Reime Jamal Shalash, Raneen Mohammed Qadah, Alham Al-Sharman, Ibrahim M. Moustafa, Tamer M. Shousha, Senthil D. Kumaran, Filippo Migliorini, Nicola Maffulli

**Affiliations:** 1 Department of Physiotherapy, College of Health Sciences, University of Sharjah, Sharjah, United Arab Emirates; 2 Neuromusculoskeletal Rehabilitation Research Group, RIMHS-Research Institute of Medical and Health Sciences, University of Sharjah, Sharjah, United Arab Emirates; 3 Sustainable Engineering Asset Management Research Group, RISE-Research Institute of Science and Engineering, University of Sharjah, Sharjah, United Arab Emirates; 4 Adjunct Faculty, Manipal College of Health Professions, Manipal Academy of Higher Education, Manipal, Karnataka India; 5 Department of Physiotherapy, Manipal College of Health Professions, Manipal Academy of Higher Education, Manipal, Karnataka India; 6 Department of Orthopaedic, Trauma, and Reconstructive Surgery, RWTH University Medical Centre, Pauwelsstraße 30, 52074 Aachen, Germany; 7 Department of Orthopedics and Trauma Surgery, Academic Hospital of Bolzano (SABES-ASDAA), 39100 Bolzano, Italy; 8 Department of Trauma and Orthopaedic Surgery, Faculty of Medicine and Psychology, University La Sapienza, Rome, Italy; 9 School of Pharmacy and Bioengineering, Keele University Faculty of Medicine, Stoke On Trent, England; 10 Queen Mary University of London, Barts and the London School of Medicine and Dentistry, Centre for Sports and Exercise Medicine,, Mile End Hospital, 275 Bancroft Road, London, E1 4DG England; 11 Faculty of Physical Therapy, Cairo University, Cairo, Egypt; 12 Department of Life Sciences, Health, and Health Professions, Link Campus University, Rome, Italy; 13 Department of Rehabilitation Sciences, Faculty of Applied Medical Sciences , Jordan University of Science and Technology, Irbid, Jordan

**Keywords:** Accelerometer, Physical activity, Validation, International Physical Activity Questionnaire (IPAQ-SF)

## Abstract

Self-reported physical activity questionnaires (e.g., International Physical Activity Questionnaire, IPAQ) are a cost-effective, time-saving, and accessible method to assess sedentary behaviour and physical activity. There are conflicting findings regarding the validity of self-reported questionnaires in comparison to accelerometer-measured data in a free-living environment. This study aimed to investigate the concurrent validity between self-reported Arabic–English IPAQ short form (IPAQ-SF) and Fibion (Fibion Inc., Jyväskylä, Finland) accelerometer-measured sedentary and physical activity time among young adults. One hundred and one young healthy adults (mean age 20.8 ± 2.4 years) filled in the IPAQ short form (IPAQ-SF) and wore the Fibion device on the anterior thigh for ≥ 600 min per day for 4–7 days. Concurrent validity between the IPAQ-SF and Fibion accelerometer for sitting, walking, moderate activity, and vigorous activity time was assessed using the Spearman correlation coefficient ($\rho$) and Bland–Altman plots. Significant weak associations between IPAQ-SF and Fibion measurements were found for total activity time ($\rho$ = 0.4; *P* < 0.001) and for the duration of walking ($\rho$ = 0.3; *P* = 0.01), moderate ($\rho$ = 0.2; *P* = 0.02), and vigorous-intensity activities ($\rho$ = 0.4; *P* < 0.001). However, $\rho$ was not significant ($\rho$ = − 0.2; *P* = 0.09) for sitting time. In addition, all the plots of the measured variables showed a proportional bias. A low association and agreement were found between self-reported IPAQ-SF scores and Fibion accelerometer measurements among young adults in the UAE. Adult sedentary and physical activity measurements should be obtained objectively with accelerometers rather than being limited to self-reported measures.

## Introduction

Physical activity (PA) plays a major role in the management and prevention of chronic diseases. However, the prevalence of physical inactivity worldwide has increased; one out of five adults around the world is inactive [1]. The Global Status Report on PA of 2022 showed that more than 80% of adolescents and 27% of adults are not physically active at levels recommended by the World Health Organization (WHO) [2]. Insufficient PA is the fourth leading risk factor for all deaths, according to the WHO [3]. The level of PA has a graded linear relationship with health status, with the most active individuals having a reduced risk of premature death [4]. Regular PA is essential to reduce the morbidity and mortality associated with chronic diseases and it provides primary and secondary prevention of these conditions [5]. Thus, encouraging an active lifestyle may help to reduce adverse health outcomes in young adults [6].

Although Arab men and women are aware of the importance of engaging in PA, several factors (e.g., sports facilities, time, gender and cultural norms, policy support, and/or hot climate) impact such knowledge, not necessarily transferring into action [7, 8]. In the United Arab Emirates (UAE), adult Emiratis and expatriates have high rates of obesity and chronic diseases (e.g., diabetes mellitus) which might result from changes in their lifestyle associated with the rapid socioeconomic transition and growth that the country has experienced [9]. Advances in transportation, modernization, overweight, and obesity were associated with reduced PA levels among UAE adults [5].

Men and non-Arab individuals have shown better self-reported PA (documented with the IPAQ-SF) than women and Arab individuals in the UAE during the COVID-19 pandemic [10]. According to a systematic review and meta-analysis by Chaabane et al., the combined prevalence of both moderate and vigorous levels of PA amongst youth in the UAE is 36.0% (95% CI = 23.9–49.9%) [11]. Young adults in the UAE spend ≈ 80% of their waking time in sedentary activities (12) and only around one-fifth of the young adults in the UAE undertook moderate PA and less than a quarter practised vigorous PA [13]. Furthermore, Emirati working women have shown a high sitting time (≈ 11 h) and low vigorous activity time (≈ 2–3 min) per 16-h day (the assumed maximum wake hours per day) [14]. These findings warrant further investigation and management of sedentary behaviour and PA of the UAE population.

PA intensity, duration, frequency, and mode can all be measured using subjective and objective methods. Subjective methods include self-report questionnaires [15, 16], such as the international PA questionnaire (IPAQ) in its long and short versions [17]. The IPAQ [17] is cost-effective, easy to administer, and easy to explain. Acceptable test–retest reliability and concurrent validity have been reported for the IPAQ-SF [18]. Subjective approaches are limited by memory, comprehension, perception, and social desirability [19]. Objective approaches minimize some measurement errors associated with subjective approaches. Objective approaches include pedometers (Fitbit, Realalt 3DTriSport Walking, etc.) and accelerometers (ActiGraph, Fibion, activPAL, etc.). Objective methods can also collect a significant quantity of data. In clinical and epidemiological research settings, the accelerometer is the most frequently used objective approach [20, 21]. Accelerometers are, however, relatively expensive and time-consuming [19, 22].

Self-reported measures underestimate sedentary time when compared to objective measures. When compared to objective measures, single-question measures from IPAQ-SF and the GPAQ resulted in a considerable underestimation of sedentary time [23]. The association between the IPAQ-SF and objective measures of activity or fitness is frequently well below acceptable thresholds. Comparing the IPAQ and accelerometer measures of PA, the IPAQ is more likely to overestimate actual PA given its limited ability to divide PA into low- and high-PA categories [24]. The IPAQ-SF compared to the device-measured PA overestimates walking time and total MET minutes while it underestimates sitting time [24]. Similarly, when PA levels were compared from the IPAQ-SF and accelerometers in older adults, sitting time was underestimated with the IPAQ-SF while moderate and vigorous PA times were overestimated [18].

A study on young adults in the UAE showed an average sitting time of around 8 h/day and high moderate-to-vigorous physical activity levels (≈ 66 ± 75 h per week [≈ 9.5 ± 10 h per day]) based on self-reported Global Physical Activity Questionnaire (GPAQ) data [25]. On the other hand, PA measured by Actigraph accelerometers worn around their waist [12] revealed that young adults in the UAE used nearly 80% (≈ 12 ± 1 h per day) of their waking time in sedentary activities and 4.4% (≈ 40 ± 20 min per day) in moderate-to-vigorous physical activity. Furthermore, PA measured with the Fibion accelerometers in Emirati working women revealed a high sitting time (≈ 11 ± 1 h), acceptable levels of moderate activity time (≈ 40 ± 18 min), and low vigorous activity time (≈ 2 ± 1 min) per 16-h day (the assumed maximum wake hours per day) on an average [14]. The Arab Teen Lifestyle Study PA questionnaire (ATLS-2) compared with Fibion accelerometer-measured data among adolescents and young adults in the UAE [26] showed a low agreement between the two methods, where the ATLS-2 underestimated sitting and PA time data compared to the accelerometer data [26]. The ATLS-2 questionnaire was specifically designed for Arab participants aged between 14 and mid-twenties [27–29], addressing the unique characteristics and activity patterns of Arab teenagers and young adults. However, studies investigating the validity of the IPAQ-SF have focused on adult and older adult populations [24]. It is evident that there is a tendency to overestimate or underestimate PA levels with self-reported measures (e.g., IPAQ, GPAQ, ATLS-2, etc.) compared to the activity monitors (e.g., accelerometers such as Actigraph, Fibion, etc.) in the UAE population and beyond [24].

Further studies are required to validate self-reported PA levels using the IPAQ-SF with accelerometer-measured sedentary and PA time in young adults employing bilingual (Arabic–English) versions to encompass a diverse range of non-Arabic and Arabic-speaking individuals from different nationalities in the UAE. The validity and reliability of the English and Arabic IPAQ-SF have been reported [30–34]. The findings of our study would be useful to conduct systematic reviews and meta-analyses on the validity of the IPAQ-SF while pooling data from other studies in this area from various countries.

The Fibion is a tri-axial thigh-worn accelerometer that measures the duration of sitting, standing, walking, cycling and PA at different intensities and associated energy expenditure [35, 36]. This device purportedly has a high storage and battery capacity and has been validated by indirect calorimetry and direct observation during a 12-h guided series of tasks such as walking, cycling, standing, and sitting [26, 36–38]. The Fibion device can assess the types and intensity of PA and the corresponding energy expenditure throughout a prolonged period with different postural adjustments [26, 35–38]. Both the Actigraph and Fibion devices demonstrated comparable reliability estimations. The Fibion devices may determine different intensities and types of PA when placed on the thigh compared to when it is worn in the trouser pocket during the 12-h working day [36]. Furthermore, the Fibion has shown good to excellent validity [35, 36] and comparable inter-monitor reliability in measuring sedentary and non-sedentary time when compared to the ActivPAL monitor [37, 39]. Therefore, this study aimed to investigate the concurrent validity between the IPAQ-SF and Fibion accelerometer sedentary and PA time among young adults in the UAE.

## Methods

### Study design

A cross-sectional observational study on healthy young adults was conducted at the College of Health Sciences, University of Sharjah, UAE. We received ethical approval from the Institutional Ethics Committee, University of Sharjah (REC-20-08-17-01).

### Study participants

One hundred and one healthy individuals (both sexes), aged from 18 to 35 years were recruited using a convenient sampling method. A sample size > 100 is recommended for the validation of questionnaires by the COSMIN guidelines [40]. Individuals had no musculoskeletal, rheumatic, cardiovascular, or systemic diseases or recent surgeries that might have impacted PA levels. Participants were recruited by posting adverts on social networking websites (e.g., Twitter, LinkedIn, Facebook), mobile apps (e.g., WhatsApp, Botim), university/school notice boards, newspapers, fliers, and/or word of mouth.

### Procedure

Before data collection, written informed consent was provided by all eligible participants. Sociodemographic, anthropometric, and other characteristics were collected from all participants. A portable stadiometer (SECA 213, SECA, Hamburg, Germany) was used to measure the participants’ height. A body composition analyser (Tanita HD-318, Tanita, Tokyo, Japan) was used to measure body composition (mass, body fat percentage, and visceral fat). Data were collected by three research assistants who were qualified physiotherapists supervised by the research team.

### PA assessment

Initially, the self-reported PA levels of participants were documented using a bilingual Arabic–English IPAQ-SF. The IPAQ-SF is used to report the time individuals spent in sitting, walking, moderate-intensity, and vigorous-intensity activities during the previous week [23]. Device-based (accelerometer) assessment of PA levels was then carried out using a Fibion device (Fibion Inc, Jyväskylä, Finland). Participants were instructed to wear the device for 7 days on the proximal third of the anterior aspect of the thigh, following the guidelines provided on the official Fibion website. The Fibion was used as the ground truth for which the IPAQ-SF questionnaire was compared against. The device was secured to the body, using an elastic strap with a Velcro attachment or a non-allergic adhesive tape supplied by the device maker [41]. When fully charged, the Fibion can measure important dimensions of PA, such as lying down, sitting, standing, and walking [37]. The Fibion Device is a 3D tri-axial accelerometer, equipped with firmware algorithms that efficiently process accelerometer data, instantly translating it into categorized activity classes and corresponding energy expenditures [42].

Data processing for Fibion data was used based on similar previous studies [35–37] performed using Microsoft Excel sheets. The data extracted from the Fibion device for each participant with their sociodemographic information were uploaded on the manufacturer's website (www.fibion.com/upload). Consequently, explicit reports regarding the PA intensity, time, and type were generated and downloaded. The data obtained and processed from CSV files contained minute-by-minute and day-by-day data. We used a bespoke data fixer tool to remove data recorded during standard night-time hours [11 pm to 7 am] for all participants [26]. This step avoided any conflation between night-time data with sedentary or upright activities. Furthermore, only participants with at least 10 h (600 min) of Fibion accelerometer-recorded data per day for 3 weekdays and 1 weekend day were included in the analysis [26]. The duration of each activity was normalized to 16 h of waking time per day to account for possible differences in Fibion device wear time amongst participants [26, 43, 44].

### Data analysis

The Shapiro–Wilk tests were used to test the normality of the data. Since the data were not normally distributed, Spearman correlation ($\rho$) was used to determine the correlation between the IPAQ-SF and Fibion data for sitting, walking, and moderate and vigorous PA time. $\rho$ was interpreted as negligible (0.00–0.10), weak (0.10–0.39), moderate (0.4–0.69), strong (0.70–0.89), or very strong (0.90–1.00) [45]. *P* values < 0.05 were considered statistically significant. All statistical analyses were performed using the IBM SPSS Statistics Version 28 (IBM Corp., Armonk, NY, USA).

The Bland–Altman plots along with 95% limits of agreement were used to identify any outliers and assess potential systematic or proportional bias. These plots depicted the mean values against the differences between the Fibion accelerometer data and the self-reported IPAQ-SF data for each outcome of interest. The 95% limits of agreement were represented as mean ± (1.96*SD), where mean and SD were derived from differences between Fibion and IPAQ-SF measurements, respectively. Moreover, linear regression analyses were performed to examine proportional bias, with the mean and difference scores of both methods used as independent and dependent variables, respectively.

## Results

One hundred and four participants took part in this study. Three participants were excluded because of technical errors in data collection. This left 101 participants for analysis. The participants were predominantly women (*n* = 71), with a mean age of 20.8 (± 2.40) years and an average body mass index (BMI) of 22.7 (± 4.73) kg/m^2^.

There was no evidence of a statistically significant correlation between self-reported IPAQ-SF and Fibion-measured sitting time ($\rho$= − 0.2; P = 0.09). Significant weak correlations between IPAQ-SF and Fibion measurements were evident for the duration of walking ($\rho$ = 0.3; *P* < 0.01), moderate ($\rho$ = 0.2; *P* < 0.02), vigorous activities ($\rho$ = 0.4; *P* < 0.001), and the total activity time ($\rho$ = 0.4; *P* < 0.001). These results are summarized in Table 1. Compared to Fibion data, participants overestimated their self-reported IPAQ-SF vigorous activity time, and they underestimated their IPAQ-SF sitting, walking and moderate PA time.

**Table 1:** Spearman correlation coefficients of IPAQ-SF and Fibion duration (*n* = 101)

Physical activity	IPAQ-SF duration Median (IQR)	Fibion duration Median (IQR)	Rho	*P*	Strength of correlation
Sitting (h)	5.0 (3.0)	9.83 (1.24)	− 0.2	0.09	Weak
Walking (min)	30.00 (45.00)	79.64 (39.86)	0.3	0.01	Weak
Moderate intensity (min)	0.00 (30.00)	36.09 (30.95)	0.2	0.02	Weak
Vigorous intensity (min)	0.00 (37.50)	0.07 (0.07)	0.4	< 0.001	Weak
Total activity time (min)	65.00	114.07	0.4	< 0.001	Weak

From the self-reported IPAQ-SF, participants were sitting for approximately 5 h a day, walking for 1 h, and spending around 20 min in moderate and vigorous PA on average. The Fibion PA data showed that participants on average spent 9 h sitting in a day, less than 2 h walking, around 30 min in moderate activity, and less than a minute in vigorous activity, on average, during 16 h of wake time per day.

**Fig. 1:**
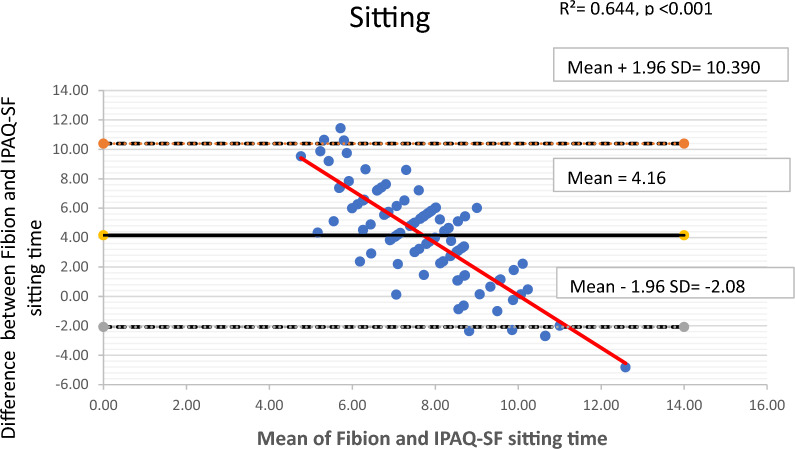
A Bland–Altman plot depicting agreement between IPAQ-SF and Fibion accelerometer-measured sitting time. The regression line appears red

**Fig. 2:**
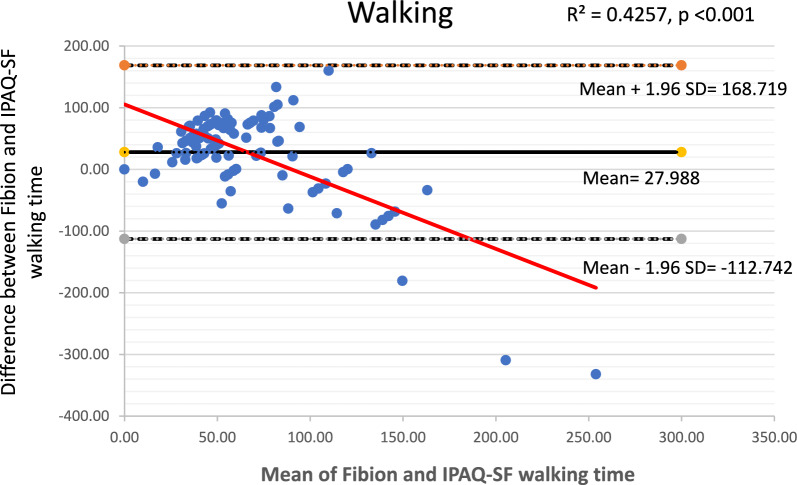
A Bland–Altman plot depicting agreement between IPAQ-SF and Fibion accelerometer-measured walking time. The regression line appears red

**Fig. 3:**
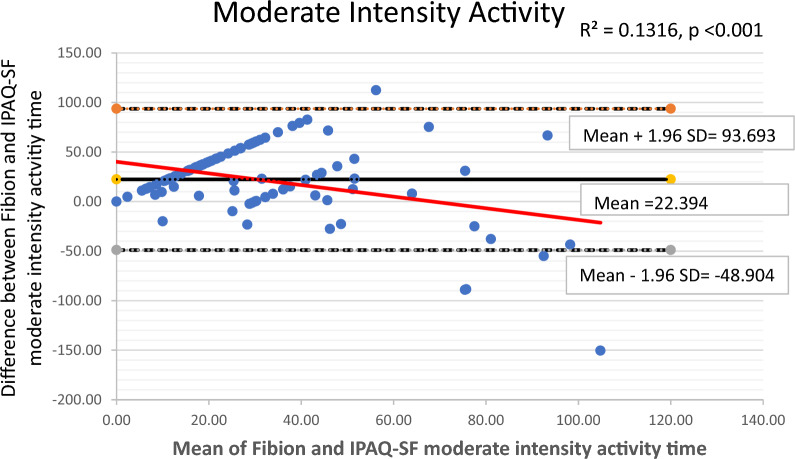
A Bland–Altman plot depicting agreement between IPAQ-SF and Fibion accelerometer-measured moderate-intensity activity time. The regression line appears red

**Fig. 4:**
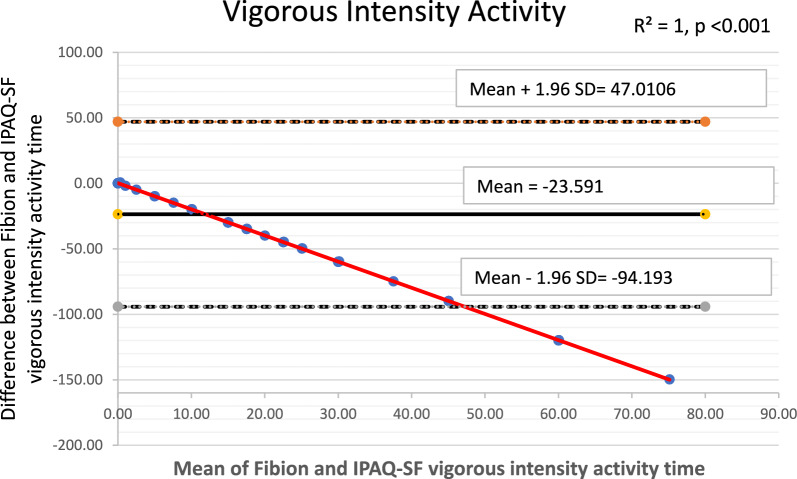
A Bland–Altman plot depicting agreement between IPAQ-SF and Fibion accelerometer-measured vigorous-intensity activity time. The regression line appears red

**Fig. 5:**
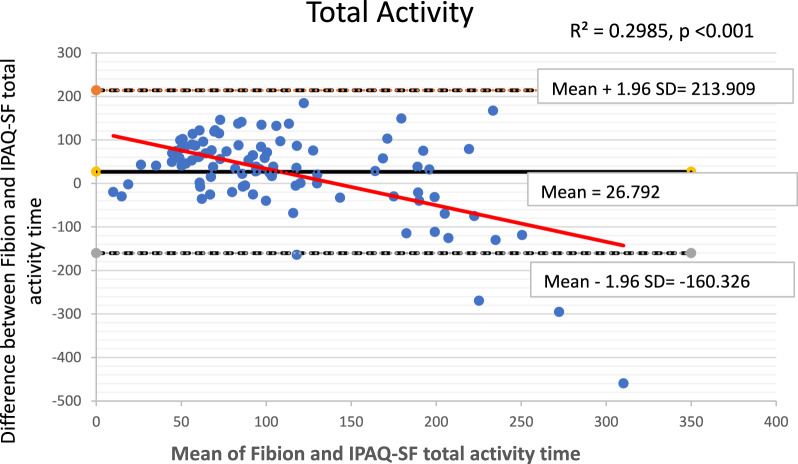
A Bland–Altman plot depicting agreement between IPAQ-SF and Fibion accelerometer-measured total activity time. The regression line appears red

The Bland–Altman plots depicting means versus differences between the Fibion and IPAQ-SF for all variables of interest are shown in Figs. 1, 2, 3, 4 and 5. A proportional bias was evident in all the plots (Figs. 1, 2, 3, 4 and 5), supported by statistically significant regression models (*P* values < 0.05). As evident from the regression lines included in the plots, an increase in the mean scores corresponded to a decrease in the difference scores between IPAQ-SF and Fibion data.

## Discussion

The present study identified a weak association between self-reported IPAQ-SF and Fibion accelerometer for sitting, walking, moderate activity and vigorous activity time. The relationships between self-reported IPAQ-SF data and PA accelerometer measures are evident when PA bouts are assessed and reported with an accelerometer [46, 47]. The accelerometer recorded more time spent undertaking PA compared to the IPAQ-SF. The self-reported IPAQ-SF underestimated sitting, walking and moderate activity duration, whereas it overestimated the vigorous activity time among young adults. These results show how subjective and objective measurements of PA in young age groups differ. Numerous studies underscore the critical significance of PA for overall health and well-being. However, despite this awareness, barriers still persist that might hinder individuals from engaging in regular PA in the UAE [7, 8]. Advocating the use of objective measures (with accelerometers) to monitor sitting and PA, our study highlights the usefulness of such methods in providing quantitative and accurate estimates compared to self-reported questionnaires (such as the IPAQ-SF). This emphasis on objective data not only enhances the validity of PA measurement, but also allows individuals to make informed decisions about sedentary behaviour and PA choices impacting their health.

Consistent with our findings, a previous systematic review found that self-reported measurements showed greater PA levels than objective assessments with accelerometers; however, sedentary behaviours were often underestimated when using self-reported measures [48]. Another systematic review, accelerometers were shown to be appropriate for continuous long-term assessments, which is not possible with self-reported measures since individuals cannot recollect or estimate the activity covered over extended periods. This accounts for the weak associations between PA levels when measured subjectively and objectively [49].

Although self-reported PA questionnaires are considered reliable [50], their association with objective measurements is only moderate, with some differences among different populations. In the current study, young adults reported fewer sedentary hours in the IPAQ-SF, but accelerometer measurements revealed greater PA duration. Self-reported measures were more valid to measure the total PA duration in older adults, but not moderate to vigorous PA duration [6]. Similarly, older adults overestimated their moderate to vigorous PA minutes in the IPAQ-LF compared to what recorded by accelerometer [51]. Another study revealed no difference in the outcomes between healthy persons and patients with orthopaedic injuries where PA levels were greater in IPAQ-SF and sitting hours were less [24]. In the obese and overweight population, the IPAQ-SF classified participants as having higher activity levels compared to objective measurements [52]. Similar to our study findings, a systematic review and a meta-analysis conducted on adults in the European union, revealed that IPAQ questionnaire provided low criterion validity measurements regarding sedentary time [50].

Self-reported data, when compared to device-based measurements, showed an under-reporting of sitting time by 4.16 h/day. The IPAQ-SF depends on respondents' ability to accurately remember and honestly report their activity and sedentary time in the previous 7 days [53]. Since self-reported activity time tends to be better recalled than overall day sitting/sedentary time, this explains the underestimation of the IPAQ-SF sitting time. Similar findings were evidenced in the previous studies comparing the IPAQ-SF and accelerometery objective measurements of PA in adults, where the IPAQ-SF considerably overestimated the time spent in vigorous PA and greatly underestimated the time spent sitting [18, 54].

By definition, the Fibion accelerometer does not depend on self-recall of PA; instead, it detects body motion, measures all 3 dimensions of PA on a minute-by-minute basis, and provides the duration and intensity of the task completed [36]. Therefore, accelerometers provide more accurate PA information and are considered a better indicator of PA since they eliminate the inherent subjectivity of self-reported questionnaires [18, 55]. However, the accelerometer may read the reclining or side-lying postures as sitting, which might explain why the Fibion-derived data included more sitting hours than the IPAQ-SF values. Although night-time data were removed from the interpreted data, it should be emphasized that the Fibion accelerometer still considers lying down awake throughout the day without moving as sitting time. Additionally, the accelerometer can perceive twisting motions while lying down and side-lying position as activity other than sleeping/sitting [56].

The duration of vigorous activity was slightly overestimated in the IPAQ-SF self-reported questionnaire. The participants may have over-reported vigorous physical levels for social-desirability-related reasons, which might be one cause for this overestimation. These findings are consistent with the findings from a study in adolescent boys, which highlighted that differences between self-reported and accelerometer-measured data are greater with higher activity levels [57]. Younger adults and individuals with higher levels of PA tend to have a more diverse range of activities, making the overall amount of moderate and vigorous activity more challenging to estimate. Furthermore, people lack a fixed mechanism for distinguishing between moderate and vigorous activity, and the perceived limit might vary greatly from person to person.

Although participants were asked to self-report activities time for a full 24-h day, the IPAQ-SF underestimated sitting, walking, and moderate activity duration, compared to the Fibion data normalized to a 16-h day. The Fibion data were normalized to a 16-h day to mitigate variations in participants' device wear time. However, these differences between the self-reported IPAQ-SF data and the normalized Fibion data were not expected to confound the correlation values reported in the study.

Our previous study investigated the validity of the Arab Teens Lifestyle Study Questionnaire 2 (ATLS-2) by comparing it to Fibion accelerometer-measured data among 131 adolescents and young adults, aged 14 years to mid-twenties, living in the UAE [26]. The current study on the validation of the IPAQ-SF questionnaire with the Fibion accelerometer data included 101 healthy individuals aged between 18 and 35 years. Some participants, but not all, participated in both projects. The present study, in concordance with the previous investigation, had confirmed the weak correlations between self-reported and Fibion-accelerometer measurements of sitting and PA times. Indeed, self-reported sitting and PA times were lower than those of Fibion-accelerometer measurements in our previous investigation on the ATLS-2 PA questionnaire and the current study on the IPAQ-SF questionnaire.

### Strengths and limitations of the study

To the best of our knowledge, this study is the first to investigate the validity of the self-reported bilingual Arabic–English version of the IPAQ-SF and Fibion accelerometers for documenting sitting and PA levels in young adults in the UAE. The IPAQ-SF serves as a subjective measure of PA, relying on individuals' recall of their activity levels over a specified period. While this reliance on memory could introduce bias into the data, particularly because of inaccuracies or variations in recall memory, the questionnaire offers significant advantages in terms of data collection for larger populations. Despite its subjective nature, the IPAQ-SF allows reporting of the duration of activity based on its context (e.g., vigorous physical activities such as heavy lifting, digging, aerobics, or fast bicycling). We employed a bilingual Arabic–English version of the questionnaire in this study, which demonstrates a concerted effort to capture data from non-Arabic and Arabic-speaking young adults in the UAE. The participants had the option to respond to either the Arabic or English versions of the questions, depending on their choice. This approach underscores the utility of the IPAQ-SF in enabling researchers to gather comprehensive data across different ethnic groups, ultimately contributing to a more nuanced understanding of self-reported PA patterns within the UAE population. Even so, it must be noted that the correlations between self-reported and accelerometer-measured sitting and PA measurements are low in the current study. Assessor-guided documentation of self-reported sedentary behaviour and PA is recommended in future studies to improve the documentation of such data.

## Conclusion

The self-reported Arabic–English IPAQ-SF questionnaire significantly underestimated sitting, walking, and moderate PA time and overestimated vigorous PA time. Therefore, the IPAQ-SF did not adequately reflect the actual sedentary and PA levels of young adults. As self-reported data underestimate sitting and PA times, accelerometer-based measures, along with self-reported PA questionnaires, are necessary when assessing PA among young individuals whenever possible to ascertain valid PA estimates.

## Data Availability

The datasets generated are under reasonable request to Ashokan Arumugam (aarumugam@sharjah.ac.ae; ashokanpt@gmail.com).

## Abbreviations

IPAQ: International Physical Activity Questionnaire
IPAQ-SF: International Physical Activity Questionnaire Short Form
GPAQ: Global Physical Activity Questionnaire
PA: Physical activity
WHO: World Health Organization
UAE: United Arab Emirates
BMI: Body mass index
ATLS-2: Arab Teens Lifestyle Study Questionnaire 2
